# Error Traps in Pediatric Neuromuscular Block

**DOI:** 10.1002/pan.70202

**Published:** 2026-04-30

**Authors:** Gabriel Soares de Sousa, Debra Faulk, Vinicius Caldeira Quintao, Niekoo Abbasian, Hans D. de Boer, Ricardo Vieira Carlos

**Affiliations:** ^1^ Discipline of Anesthesiology, Faculdade de Medicina Universidade de São Paulo São Paulo Brazil; ^2^ Instituto da Criança e do Adolescente, Hospital das Clínicas HCFMUSP, Faculdade de Medicina Universidade de São Paulo São Paulo Brazil; ^3^ Department of Pediatrics, Faculdade de Medicina Universidade de São Paulo São Paulo Brazil; ^4^ Serviços Médicos de Anestesia, Hospital Sírio‐Libanês São Paulo Brazil; ^5^ Department of Anesthesiology University of Colorado Aurora Colorado USA; ^6^ Division of Pediatric Anesthesiology Children's Hospital Colorado Aurora Colorado USA; ^7^ Department of Anesthesiology Cincinnati Children's Hospital Medical Center Cincinnati Ohio USA; ^8^ International Network to Improve Perioperative Care (INIPC) Academy Groningen the Netherlands

**Keywords:** neostigmine, neuromuscular blocking agents, neuromuscular monitoring, pediatric anesthesia, sugammadex

## Abstract

**Background:**

Neuromuscular blocking agents are essential for safe pediatric anesthesia but remain a frequent source of preventable morbidity when misused, inadequately monitored, or incompletely reversed. Children, particularly neonates and infants, are especially vulnerable to residual neuromuscular block due to developmental pharmacological variability and limited physiological reserve.

**Aims:**

To describe common and preventable “error traps” in pediatric neuromuscular block management and to highlight strategies to improve safety across the perioperative continuum.

**Methods:**

This narrative review synthesizes current evidence and clinical practice patterns to identify recurrent pitfalls in the use, monitoring, reversal, and postoperative management of neuromuscular blocking agents in children.

**Results:**

Four major error traps were identified: omission of neuromuscular blocking agents when optimal intubating conditions are required; failure to use quantitative neuromuscular monitoring; inappropriate or mistimed pharmacological reversal; and failure to recognize and treat residual paralysis. These errors contribute to a persistently high incidence of residual neuromuscular block and are associated with increased risk of postoperative respiratory complications. Developmental pharmacokinetic and pharmacodynamic variability further amplifies these risks in pediatric populations.

**Conclusions:**

Avoidance of these error traps requires systematic application of evidence‐based practices, including routine use of quantitative neuromuscular monitoring and objective confirmation of recovery with a train‐of‐four ratio ≥ 0.9 prior to extubation. Embedding these principles into clinical practice is essential to improving safety in pediatric anesthesia.

## 1 Introduction

Ensuring patient safety during pediatric anesthesia hinges on the meticulous management of neuromuscular block. Profound physiological and pharmacological differences between children and adults, combined with the high inherent risks of airway management in younger patients, create unique challenges in this field [1]. Despite advances in monitoring and pharmacology, several recurrent pitfalls, here referred to as “Error Traps”, continue to compromise perioperative safety [2]. These error traps represent situations that predispose to preventable complications, most often arising from flawed protocols, cognitive biases, or system‐level deficiencies rather than individual negligence [3].

Neuromuscular blocking agents (NMBA) remain indispensable in modern pediatric anesthesia, facilitating tracheal intubation, ensuring immobility during surgery, and optimizing surgical conditions [4]. In neonates and infants, where rapid oxygen desaturation and airway difficulties are common, the use of NMBA is particularly recommended whenever maintenance of spontaneous ventilation is not required [5]. However, NMBA administration in these populations is complicated by substantial physiological variability and persistent gaps in clinical practice. Misuse, inadequate monitoring, and incomplete reversal frequently contribute to residual neuromuscular block (RNMB), a well‐recognized yet under‐addressed cause of postoperative morbidity [4].

**FIGURE 1:**
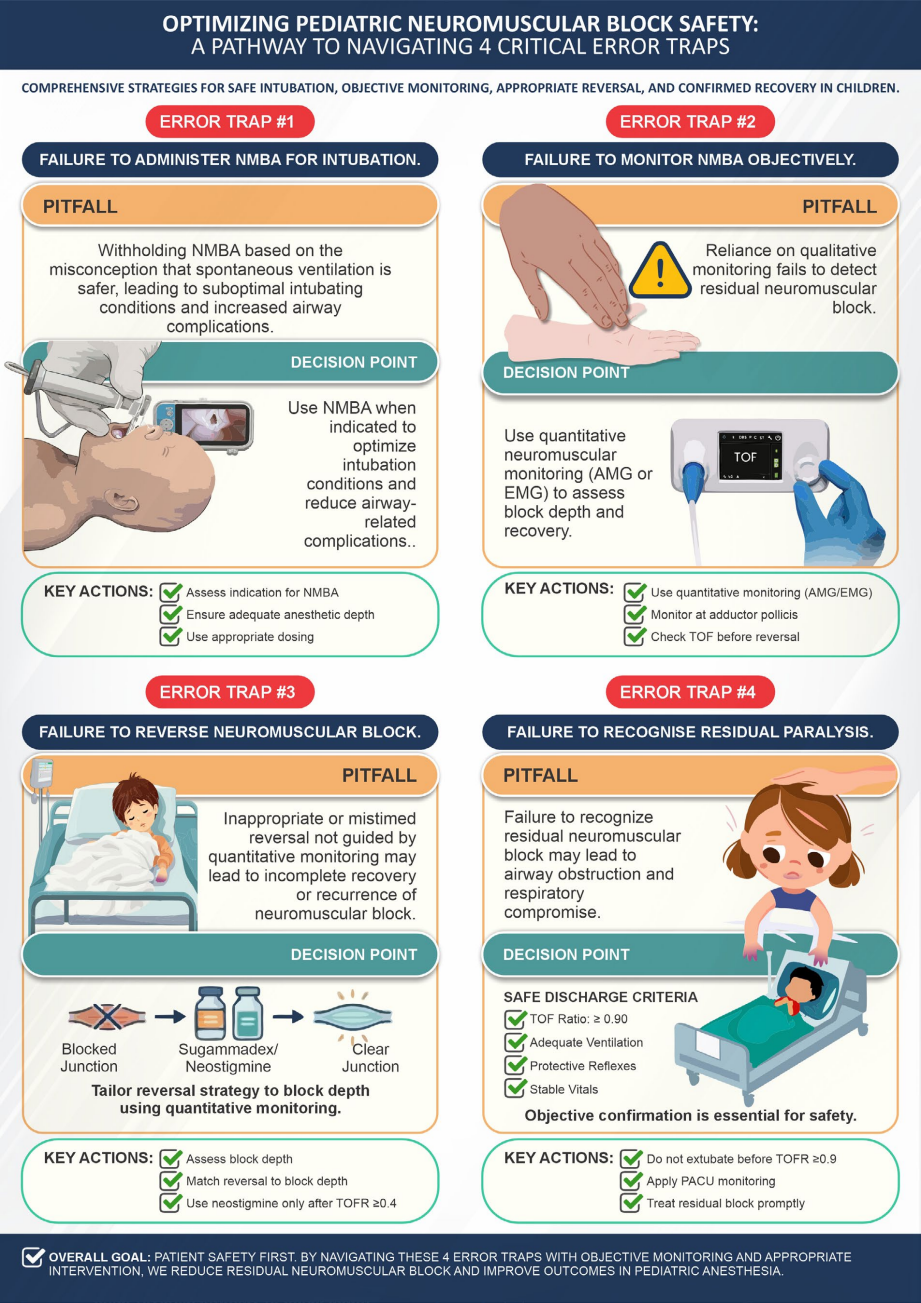
Optimizing pediatric neuromuscular block safety: A pathway to navigating four critical error traps. NMBA, neuromuscular blocking agent; TOF, train‐of‐four; AMG, acceleromyography; EMG, electromyography; PACU, post‐anesthesia care unit.

This review delineates four major error traps in pediatric and neonatal neuromuscular block management, structured along the chronological phases of a typical anesthetic: induction, maintenance, emergence, and post‐anesthesia recovery, highlighting where clinical vigilance, quantitative monitoring, and adherence to evidence‐based principles are essential to prevent avoidable adverse outcomes in this vulnerable population (Figure 1).

## 2 Overview of NMBA

NMBA are classified based on their mechanism of action at the neuromuscular junction (Table 1):

**TABLE 1:** Overview of commonly used neuromuscular blocking agents in pediatrics.

NMBA	Primary class	Key characteristics in pediatrics	Essential precautions/contraindications
Succinylcholine	Depolarizing	Ultrarapid onset, ultrashort duration. Metabolism: plasma cholinesterase.	Contraindication in conditions causing AChR upregulation (e.g., severe burns after 24 h, prolonged immobilization, sepsis, muscular dystrophy) due to risk of life‐threatening hyperkalemia and cardiac arrest. Risk of masseter muscle rigidity and MH susceptibility.
Rocuronium	Aminosteroid	Intermediate duration, rapid onset (most common ND‐NMBA used in pediatric difficult intubation). Metabolism: primarily hepatic and biliary clearance.	Duration is prolonged by hepatic or renal dysfunction. Reversible by sugammadex.
Vecuronium	Aminosteroid	Intermediate duration, slower onset than rocuronium. Metabolism: predominantly hepatic with formation of active metabolite (3‐desacetyl‐vecuronium). Renal excretion contributes to clearance.	Prolonged effect in hepatic or renal dysfunction. Active metabolite may accumulate during prolonged infusion. Reversible with sugammadex or anticholinesterases (e.g., neostigmine).
Cisatracurium	Benzylisoquinolinium	Intermediate duration. Eliminated primarily via organ‐independent Hofman degradation.	Predictable clearance independent of renal or hepatic failure. Does not induce histamine release. Reversible only by anticholinesterases (e.g., neostigmine).
Sugammadex	Selective reversal agent	Rapid, predictable reversal of steroidal NMBA.	FDA‐approved for pediatric patients from birth. Drug interaction: may reduce efficacy of hormonal contraceptives in postmenarchal females.

Abbreviations: AchR, acetylcholine receptor; FDA, Food and Drug Administration; MH, malignant hyperthermia; ND‐NMBA, non‐depolarizing neuromuscular block agent; NMBA, neuromuscular block agents.

Depolarizing agents: Succinylcholine is the only depolarizing NMBA still in clinical use. Acting as an acetylcholine receptor (AChR) agonist, it produces persistent depolarization of the motor endplate. Succinylcholine is characterized by its ultrarapid onset and short duration of action, due to rapid hydrolysis by plasma cholinesterase (butyrylcholinesterase) [6].

Non‐depolarizing NMBA (ND‐NMBA): These compounds competitively antagonize the binding of acetylcholine at post‐junctional AChRs. ND‐NMBA are structurally classified into aminosteroidal and benzylisoquinolinium compounds, whose chemical structures determine their elimination pathways and influence their reversibility with agents such as sugammadex or neostigmine [6].

- Aminosteroidal agents: include rocuronium and vecuronium. These agents rely predominantly on hepatic and renal clearance for elimination.
- Benzylisoquinolinium agents: include cisatracurium and atracurium. Their elimination is largely independent of organ function, primarily through spontaneous chemical degradation known as Hofmann elimination. This process depends on physiological temperature and pH rather than hepatic or renal metabolism.
- Other ND‐NMBA, such as pipecuronium and mivacurium, remain available in certain regions outside the United States and should be considered according to local practice patterns and pharmacologic profiles.

## 3 NMBA in Pediatrics

Pediatric patients demonstrate marked inter‐individual variability in their response to NMBA [6]. In neonates and infants, the combination of increased neuromuscular sensitivity (reflecting developmental differences in AChR density and subunit composition at the neuromuscular junction) together with immature hepatic and renal clearance and altered body composition, results in a more potent and prolonged response to ND‐NMBA, despite a larger volume of distribution [1]. Conversely, children aged 2–11 years exhibit relative resistance to these agents, requiring higher doses to achieve and maintain comparable levels of block and demonstrating faster spontaneous recovery due to increased clearance and mature neuromuscular transmission [6].

## 4 The Critical Role of Monitoring and Antagonism

The most significant risk associated with NMBA administration is RNMB, defined as a train‐of‐four (TOF) ratio < 0.9, with an incidence of up to 48% in pediatric audits [7]. RNMB in children is directly associated with postoperative complications such as upper airway obstruction, pulmonary aspiration, and hypoxemia [8].

To mitigate this risk, major professional society guidelines, including the American Society of Anesthesiologists (ASA) and the European Society of Anaesthesiology and Intensive Care (ESAIC), strongly recommend that quantitative neuromuscular monitoring (acceleromyography [AMG] or electromyography [EMG]) be used throughout all phases of anesthesia whenever NMBA are administered [9, 10]. Pediatric‐specific ESAIC recommendations have recently been approved and are anticipated to further reinforce these standards [11]. Relying solely on clinical signs or subjective assessment (e.g., qualitative peripheral nerve stimulation) is demonstrably inaccurate and fails to detect RNMB when the TOF ratio lies between 0.4 and 0.9 [4].

The introduction of sugammadex has provided a highly effective means of reversal of neuromuscular block induced by aminosteroidal agents, enabling faster and more predictable recovery than neostigmine, even from deep block [12]. However, this success has paradoxically led to reduced vigilance in quantitative monitoring, particularly among less experienced practitioners who may regard sugammadex as a complete safety net [13]. Failure to employ objective monitoring undermines safe practice by obscuring accurate titration of both NMBA and reversal agents.

## 5 Error Trap #1—Induction Phase: Failure to Administer NMBA for Optimal Intubation and Ventilation Conditions

### 5.1 Overview

NMBA are integral to pediatric anesthesia, facilitating tracheal intubation, enabling controlled ventilation, and optimizing surgical conditions [4]. Despite their well‐established efficacy, reluctance to administer NMBA during induction, particularly in infants and in children with perceived difficult airways, remains common [14]. This practice, usually based on historical concerns or misconceptions, has been associated with increased airway reactivity, hypoxemia, and airway trauma, as well as lower first‐pass success rates [15].

### 5.2 Underlying Causes and Misconceptions

#### 5.2.1 The Assumption of Safety Through Spontaneous Ventilation

Maintaining spontaneous ventilation during induction has traditionally been advocated for children with anticipated difficult airways, under the assumption that it preserves oxygenation should intubation fail [14]. Contemporary evidence indicates that this approach may increase rather than reduce risk. Airway instrumentation in a lightly anesthetized child without full relaxation frequently provokes protective airway reflexes, laryngospasm, and bronchospasm. Data from the *Pediatric Difficult Intubation* (*PeDI*) *Registry* demonstrated that spontaneous‐ventilation techniques were associated with approximately twice the odds of airway complications compared with controlled ventilation, primarily owing to airway reactivity [14]. Non‐severe events, such as hypoxemia, occurred in 22.5% of cases, compared with 13.3% of cases, respectively.

#### 5.2.2 Over‐Reliance on Deep Anesthesia Alone

Some practitioners attempt to achieve sufficient relaxation solely by deepening anesthesia with volatile or intravenous agents, or both [14]. This strategy is physiologically unreliable and may result in cardiovascular instability or delayed recovery [2]. Anesthetic depth attenuates reflexes but does not predictably abolish skeletal muscle tone [1]. The concurrent use of a NMBA provides controlled, reversible relaxation, allowing lighter, more stable anesthetic maintenance [16]. Clinical studies demonstrate that combining adequate anesthetic depth with neuromuscular block yields smoother induction, reduced sympathetic activation, and improved hemodynamic stability [17].

#### 5.2.3 Inadequate Dosing or Inappropriate Agent Selection

Suboptimal dosing or inappropriate NMBA selection can negate the intended benefit [6]. Sub‐therapeutic dosing (e.g., rocuronium < 0.6 mg kg^−1^) may result in incomplete relaxation and poor laryngoscopy view, whereas use of depolarizing agents such as succinylcholine in contraindicated situations introduces avoidable risks [18]. Intermediate‐acting ND‐NMBA, particularly rocuronium and cisatracurium, are commonly used in pediatric practice. However, their onset and recovery profiles can show considerable inter‐individual variability and are influenced by age, especially in infants [19]. Onset is typically faster in infants, and duration may be prolonged under volatile anesthesia [20]. Understanding these developmental pharmacodynamic differences allows appropriate titration and timing.

#### 5.2.4 Limited Drug Availability

In low‐resource settings, the absence of certain NMBA may compel clinicians to proceed without pharmacological relaxation [20]. This represents a system‐level rather than an individual failure. Ensuring the availability of intermediate‐acting agents such as rocuronium, atracurium, or cisatracurium is therefore a fundamental patient safety requirement. Historically, long‐acting agents including pancuronium, doxacurium, and pipecuronium were associated with higher rates of RNMB. However, the introduction of intermediate‐acting NMBA did not, by itself, eliminate the incidence of RNMB when these agents replaced long‐acting drugs in routine practice [8]. Safe use therefore depends not on presumed predictability of recovery, but on appropriate dosing, vigilance, and access to quantitative neuromuscular monitoring [6].

Another frequent misconception is that readiness for intubation is purely time‐dependent, based on waiting a predetermined interval (e.g., 60–120 s) after NMBA administration. In reality, onset of adequate laryngeal relaxation varies substantially according to age, dose, cardiac output, and individual pharmacodynamic variability. Objective assessment of neuromuscular effect, when available, is preferable to reliance on fixed time intervals, as incomplete laryngeal paralysis may result in suboptimal intubating conditions and airway reactivity [21, 22].

### 5.3 Clinical Impact

Children possess limited physiological tolerance to airway obstruction or apnea [2]. High oxygen consumption and low functional residual capacity predispose to rapid desaturation, and bradycardia or cardiac arrest may occur within seconds of inadequate ventilation [16]. Intubation without sufficient neuromuscular relaxation often requires multiple attempts and prolonged airway manipulation, increasing mucosal trauma, edema, and subsequent oxygen desaturation [14]. Inadequate anesthetic depth further heightens airway reflexes and autonomic responses, which can be particularly hazardous in children with cardiac or neurological disease [23].

Conversely, relaxation of upper airway musculature decreases resistance, facilitates tidal‐volume delivery at lower inspiratory pressures, and rapidly relieves reflex glottic closure or laryngospasm, preventing the hypoxemia–bradycardia cascade. In addition, relaxation of intercostal and abdominal muscles enhances thoraco‐abdominal compliance and synchrony with positive‐pressure ventilation, resulting in more stable and predictable gas exchange, particularly in infants [18].

### 5.4 Prevention and Best Practice

These risks are largely preventable through appropriate pharmacological relaxation when maintenance of spontaneous ventilation is not required. Neuromuscular block mitigates the adverse effects of airway stimulation by improving intubation and ventilation conditions [24]. Meta‐analytic data indicate that NMBA use increases the likelihood of excellent intubating conditions (risk ratio ≈1.4) and reduces the risk of unacceptable conditions by more than half (risk ratio ≈0.35) [13]. Its deliberate omission should therefore be recognized as a preventable error with clear physiological and procedural consequences [5].

## 6 Error Trap #2—Maintenance Phase: Failure to Monitor Neuromuscular Block in Children

### 6.1 Overview

Accurate monitoring of neuromuscular block is fundamental to the safe administration of NMBA in pediatric anesthesia [25]. Failure to appropriately monitor both the depth and recovery of neuromuscular block constitutes a critical error trap, predisposing patients to RNMB and its sequelae [26]. Although the association between RNMB and postoperative pulmonary complications is well established in adults, similar patterns have not been well established in children [9]. Nevertheless, in pediatric patients, pharmacokinetic and pharmacodynamic variability, coupled with anatomical and physiological differences, may plausibly increase the risck of adverse respiratory consequences. Reliance on subjective assessment or qualitative monitoring, therefore, poses substantial and preventable risk [27].

### 6.2 Underlying Causes and Misconceptions

#### 6.2.1 Reliance on Clinical Signs

Clinical indicators of recovery, such as spontaneous ventilation, sustained head lift, or limb movement, are notoriously unreliable for detecting residual paralysis [7]. These signs may reappear when the train‐of‐four ratio (TOFR) is as low as 0.4, falsely reassuring clinicians of adequate recovery [28]. Moreover, young children cannot cooperate with such assessments, further diminishing their reliability.

#### 6.2.2 Limitations of Qualitative Monitoring

A frequent misconception in pediatric anesthesia is that monitoring neuromuscular block is unnecessary because pharmacokinetics is assumed to be predictable [13]. This notion disregards both physiological variability and developmental pharmacology. Infants and children exhibit substantial differences in NMBA onset, potency, and elimination compared with adults. Drug distribution and metabolism are highly age‐dependent, influenced by total body water, hepatic enzyme maturation, and renal clearance [6]. Even within pediatric subgroups, variability can exceed 50% for time‐to‐peak effect and recovery duration [29]. Consequently, identical doses can produce markedly different block depths and recovery profiles among patients.

Qualitative monitoring with a peripheral nerve stimulator, based on visual or tactile assessment of TOF fade, remains common in pediatric anesthesia but is inherently subjective and unreliable [30]. Human perception cannot detect fade once the TOFR exceeds approximately 0.4, leaving a clinically significant range of RNMB unnoticed [4]. Furthermore, monitoring at inappropriate sites, particularly facial muscles such as the corrugator supercilii, often chosen when the arms are tucked, tends to overestimate recovery, as these muscles are more resistant to neuromuscular block than the adductor pollicis muscle [31]. This limitation has been associated with a five‐fold increased risk of RNMB [26].

#### 6.2.3 The “Sugammadex Effect”

The availability of sugammadex has inadvertently reduced vigilance in monitoring, creating a perception that objective measurement is unnecessary [13]. However, failures related to dosing and administration have been reported when sugammadex was used without quantitative guidance, frequently due to under‐dosing or unrecognized deep block [32]. Objective confirmation of recovery remains essential regardless of the reversal method.

### 6.3 Clinical Impact

Objective, quantitative neuromuscular monitoring is the only reliable method to confirm adequate recovery, defined as a TOFR ≥ 0.9, preferably measured at the adductor pollicis muscle [25]. Among quantitative neuromuscular monitoring modalities, EMG and AMG are the most widely used in pediatric anesthesia [33]. Their key technical features and clinical considerations are summarized in Table 2. Multiple international guidelines strongly discourage reliance on clinical or qualitative assessments [9, 10]. AMG remains widely available and validated in children when free thumb movement is possible. However, its use in neonates and infants presents practical challenges, as many sensors were originally designed for adult anatomy. Small thumb size, limited excursion, and frequent limb tucking during pediatric surgery may compromise signal acquisition and increase variability. Inadequate fixation, poor calibration, or failure to normalize baseline values can further contribute to measurement error, including TOFR overshoot. Although pediatric‐specific sensors have improved feasibility, careful setup and interpretation remain essential. At the same time, EMG, which can function when arms are tucked, has emerged as an option for quantitative monitoring when hand motion is restricted or may be inaccessible, which is commonly the case with children [34]. EMG eliminates the need for facial monitoring and avoids baseline drift, showing strong agreement with AMG and mechanomyography for TOFR assessment [33]. Several portable, user‐friendly EMG devices are now validated for pediatric use.

**TABLE 2:** Quantitative neuromuscular monitoring modalities in children.

Modality	Mechanism/description	Advantages	Limitations	Examples/commercial devices
Electromyography	Measures compound muscle action potentials evoked by peripheral nerve stimulation.	Highly accurate; unaffected by limb positioning; effective even when arms are tucked; pediatric‐sized sensors available.	Susceptible to electrical interference; may exhibit baseline drift or TOFR *overshoot* (clinically irrelevant); higher cost.	*TetraGraph* (Senzime), *NMT Module* (Philips IntelliVue), *Carescape NMT*(GE Healthcare), *TwitchView* (Blink Device Company)
Acceleromyography	Measures acceleration of muscle movement (F = m × a) using piezoelectric sensors, typically on the thumb.	Widely available and validated in children; portable and user‐friendly.	Requires free thumb movement; prone to baseline drift and TOFR *overshoot* (“staircase phenomenon”) if not properly calibrated and normalized.	*ToFscan* (IDMED), *TOF‐Watch SX* (Organon), *Carescape NMT* (GE Healthcare).
Mechanomyography	Measures isometric force of muscle contraction in response to nerve stimulation.	Historically the research gold standard; provides direct measurement of muscle force.	Bulky and impractical for clinical use.	Research‐only systems (custom‐built).
Kinemyography	Detects bending of a piezoelectric sensor placed in the thenar webspace, proportional to muscle contraction.	Compact; easy to use.	Requires unimpeded thumb movement; limited clinical validation.	*E‐NMT Module* (Datex‐Ohmeda, legacy systems).

Abbreviation: TOFR, train‐of‐four ratio.

[Correction added on 22 May 2026 after first online publication: The text in the 4th column of the first row was corrected].

### 6.4 Prevention and Best Practice

Knowledge gaps among anesthesiologists regarding neuromuscular monitoring principles remain a significant barrier to safe practice. Surveys of pediatric anesthesia providers demonstrate that just over half assess neuromuscular function when using NMBA [13]. Institutional protocols that fail to mandate quantitative monitoring further perpetuate this deficiency. Moreover, the limited availability of pediatric‐specific recommendations from international societies regarding pediatric monitoring contributes to variability and underuse [9].

To mitigate the risks associated with inadequate monitoring of neuromuscular block, a multifaceted approach is required. Routine quantitative monitoring, preferably using AMG or EMG, should be mandatory for all children receiving NMBA [4]. Monitoring should be standardized at the ulnar nerve–adductor pollicis muscle site, as facial muscle assessment consistently overestimates recovery [35]. In parallel, education and institutional policy must reinforce the limitations of qualitative methods, promote proficiency with quantitative technologies, and formalize protocols that mandate their routine use in pediatric anesthetic practice.

## 7 Error Trap #3—Emergence Phase: Failure to Reverse Neuromuscular Block in Children

### 7.1 Overview

The safe and effective reversal of neuromuscular block is a cornerstone of modern pediatric anesthesia [4]. Incomplete or omitted reversal exposes children to RNMB, which is associated with upper‐airway obstruction, hypoventilation, aspiration, and postoperative pulmonary complications [8]. Despite decades of evidence, RNMB continues to occur in up to 30%–50% of children at the time of tracheal extubation, even when a reversal agent is administered [7]. This persistent problem represents not a pharmacological failure, but a system failure, rooted in misconceptions about predictability, overreliance on clinical impression, and incomplete understanding of pediatric pharmacokinetics and pharmacodynamics [36].

### 7.2 Underlying Causes and Misconceptions

A common misconception in pediatric anesthesia is that regular dosing and spontaneous recovery provide reliable and predictable reversal of neuromuscular block [20]. In reality, substantial pharmacokinetic and pharmacodynamic variability exists among infants and children, influenced by differences in body water composition, hepatic enzyme maturation, and renal clearance [6]. “Standard” or fixed doses extrapolated from adult data frequently result in overdosing in neonates and infants [29]. Rocuronium, for instance, demonstrates a larger apparent volume of distribution but slower clearance in younger children, prolonging its effect, while benzylisoquinolinium agents such as cisatracurium exhibit delayed elimination under sevoflurane anesthesia [37]. Even with agents considered “intermediate‐acting,” RNMB may persist at the end of brief procedures, particularly when volatile anesthetics, repeated boluses, or concomitant pharmacologic and physiologic factors that potentiate neuromuscular block are present [8]. Such factors include magnesium administration, certain antibiotics, electrolyte disturbances, and acid–base or temperature abnormalities. Magnesium, for example, potentiates neuromuscular block by inhibiting presynaptic calcium influx and reducing acetylcholine release, thereby prolonging both onset and recovery [17]. Quantitative monitoring studies have shown that up to 16% of children under 3 years of age had a TOFR < 0.9 after a single 0.1 mg kg^−1^ dose of cisatracurium—well beyond its expected clinical duration [20].

### 7.3 Clinical Impact

#### 7.3.1 Neostigmine: Incomplete and Time‐Dependent

Neostigmine, an acetylcholinesterase inhibitor, remains the most widely used reversal agent worldwide due to its low cost and broad availability. However, its pharmacological profile imposes important limitations. Because of its ceiling effect, doses above 0.07 mg·kg^−1^ provide no additional benefit and only increase muscarinic adverse effects such as bradycardia, salivation, and bronchospasm [38]. Neostigmine is ineffective in deep neuromuscular block and should be administered only once partial spontaneous recovery is evident (TOFR ≥ 0.4); when given too early, it can paradoxically worsen transmission and prolong weakness [17]. Its onset is variable, with full clinical recovery typically requiring 10–20 min after administration, making accurate timing essential before extubation. Even with correct dosing, quantitative studies show that up to 30% of children remain incompletely reversed [8]. These pharmacodynamic constraints underscore the necessity of quantitative neuromuscular monitoring to precisely guide reversal dosing and timing, as empirical approaches based solely on clinical signs or elapsed time fail to capture the significant interindividual variability in drug effect and recovery kinetics, thereby increasing the risk of RNMB.

#### 7.3.2 Sugammadex: Rapid but Not Infallible

Sugammadex, a selective relaxant binding agent that encapsulates aminosteroidal NMBA, such as rocuronium and vecuronium, enables rapid and generally predictable reversal across a broad spectrum of neuromuscular block depths. When administered at 2 mg·kg^−1^ for moderate block (train‐of‐four count [TOFC] ≥ 2) or 4 mg·kg^−1^ for deep block (post‐tetanic count [PTC] ≥ 1 with TOFC = 0), recovery to a TOFR ≥ 0.9 typically occurs within a few minutes. However, clinically relevant outliers have been reported, and reliance on elapsed time alone is insufficient to confirm full recovery. Pediatric randomized trials have demonstrated faster and more reliable reversal with sugammadex compared with neostigmine, with fewer cardiovascular or secretory side effects [39]. Nevertheless, reappearance of neuromuscular block and RNMB has been described, particularly in infants receiving subtherapeutic dosing or in cases of rapid redistribution of rocuronium [32]. Because reversal depends on stoichiometric complexation, an inadequate molar ratio of sugammadex to NMBA may permit unbound relaxant to re‐emerge in plasma, resulting in RNMB [32]. For very deep block (e.g., PTC = 0), evidence‐based dosing is less clearly defined, reinforcing the need for quantitative monitoring to guide dosing and confirm recovery rather than relying on empirical dosing strategies. Pharmacokinetic studies indicate that infants exhibit larger apparent volumes of distribution and slower clearance, necessitating cautious dosing and extended post‐reversal observation. Moreover, as sugammadex–NMBA complexes are renally excreted, impaired renal function may prolong exposure or permit recirculation of active drug [40]. These pharmacologic and physiologic factors explain the occasional occurrence of delayed reappearance of neuromuscular block, often within 15–60 min after apparent recovery, and underscore the need for quantitative confirmation of neuromuscular function and vigilant postoperative monitoring [32]. Importantly, the 16 mg·kg^−1^ regimen approved in adults for immediate reversal after rocuronium 1.2 mg·kg^−1^ has not been systematically studied in pediatric patients and should not be used routinely outside carefully considered emergency circumstances [39].

#### 7.3.3 Erratic or Unexpected Effects and Systemic Barriers

Both neostigmine and sugammadex have been associated with paradoxical or unpredictable outcomes when used without adequate monitoring or understanding of their pharmacodynamics. Administering neostigmine in the absence of RNMB can transiently impair neuromuscular transmission due to excess acetylcholine at the junction, manifesting as paradoxical weakness [17]. Similarly, case reports have described transient decreases in twitch response following low or partial sugammadex dosing, attributed to incomplete complexation or redistribution phenomena [41]. Reappearance of neuromuscular block, despite apparently adequate dosing, has also been documented, presenting as ventilatory distress and cyanosis 30–60 min after apparent full recovery, resolving only after an additional sugammadex bolus [40]. These observations demonstrate that pharmacological reversal with the recommended doses alone cannot guarantee sustained recovery. Only objective quantitative monitoring can confirm complete restoration of neuromuscular function and ensure long‐term safety. Beyond pharmacological factors, economic and logistical barriers continue to limit optimal practice. Sugammadex remains costly in many countries and may not be routinely stocked, fostering overreliance on neostigmine even when pharmacologically suboptimal [42]. Additionally, cognitive biases introduced by the perceived reliability of sugammadex have paradoxically fuelled complacency, creating an illusion of safety that discourages routine monitoring, despite documented failures [13]. Reversal should not be considered complete until a TOFR ≥ 0.9 is objectively confirmed.

### 7.4 Prevention and Best Practice

1. Always employ quantitative monitoring (preferably EMG or AMG) to guide timing and confirm recovery.
2. Match the reversal agent and dose to the NMBA type and block depth.
  ○ Neostigmine: only after spontaneous recovery (TOFR ≥ 0.4).
  ○ Sugammadex: dose 2–4 mg·kg^−1^ proportional to block depth. Higher doses (e.g., 16 mg·kg^−1^) have not been studied in pediatric patients. Be aware of rare but clinically significant bradycardia, particularly following rapid intravenous boluses or higher doses. Ensure continuous hemodynamic monitoring and readiness to treat with anticholinergic agents or epinephrine if needed.
  ○ Avoid time‐based reversal. Decisions should be guided by objective evidence of neuromuscular recovery.
3. Confirm TOFR ≥ 0.9 before extubation.
4. Observe high‐risk patients post‐reversal (infants, renal impairment, heavy NMBA dosing) for early signs of respiratory compromise.
5. Promote education and standardization through institutional policy, simulation, and audit feedback.

## 8 Error Trap #4—Post‐Anesthesia Care Phase: Failure to Recognize and Treat RNMB in Children

### 8.1 Overview

RNMB, if unrecognized and untreated, remains a major contributor to postoperative morbidity in pediatric anesthesia. Defined quantitatively as a TOFR < 0.9, RNMB leads to airway obstruction, hypoventilation, aspiration, and hypoxemia [26]. Despite advances in monitoring and reversal pharmacology, RNMB persists in up to 30%–50% of children at extubation [7]. The failure to promptly identify and treat RNMB represents the final, and often most dangerous, link in the chain of neuromuscular‐management error traps.

### 8.2 Underlying Causes and Misconceptions

The incidence of RNMB in children mirrors adult rates and remains unacceptably high. Prospective audits report TOFR < 0.9 in approximately 28% of cases overall and up to 48% immediately prior to extubation, with incomplete recovery persisting in the post‐anesthesia care unit (PACU) [8]. Children's unique physiology amplifies the harm caused by even minimal residual weakness. Even mild residual weakness (TOFR 0.4–0.9) can impair airway stability and ventilatory mechanics. Clinical recognition is often delayed because children cannot articulate dyspnea or weakness, and postoperative agitation or restlessness may be misinterpreted as pain or emergence delirium, prompting administration of sedatives or opioids that exacerbate respiratory depression. RNMB therefore contributes to a preventable cascade of hypoventilation, hypoxemia, and bradycardia, which can rapidly progress to cardiac arrest, especially in neonates and small infants [8].

RNMB rarely results from a single misstep but rather from the combined effect of insufficient intraoperative monitoring and suboptimal reversal practices [36]. In many centers, the continued reliance on clinical signs or qualitative peripheral nerve stimulation leads to overestimation of recovery once the TOFR exceeds approximately 0.4, leaving a “blind zone” of unrecognized paralysis (Error Trap 2). This lack of quantitative confirmation is compounded by incomplete or poorly timed pharmacological reversal (Error Trap 3). Neostigmine, although widely used, does not reliably or rapidly reverse deep neuromuscular block, as meaningful antagonism requires partial spontaneous recovery before administration. When given during profound block, recovery may consist of a prolonged period of spontaneous return of neuromuscular function before neostigmine‐assisted acceleration becomes evident, often requiring 10 min or longer for adequate recovery. Premature administration, particularly in the absence of RNMB, may paradoxically impair neuromuscular transmission and transiently worsen weakness [26]. Sugammadex, despite offering rapid and reliable reversal, may fail when underdosed, given late, or in infants with altered pharmacokinetics or renal immaturity [12]. Together, these failures reflect not isolated clinical lapses but systemic deficiencies in education, protocol adherence, and resource availability.

### 8.3 Clinical Impact

Recognition of RNMB must be followed by timely and appropriate management to prevent re‐intubation, respiratory distress, and prolonged hospitalization [38]. Treatment combines pharmacological reversal and supportive measures. When partial recovery is evident on quantitative monitoring, such as the presence of four TOF responses with residual fade, neostigmine (typically 0.02–0.05 mg kg^−1^ with appropriate antimuscarinic co‐administration) may be administered, with dosing guided by block depth and clinical context. Because of its ceiling effect and slower onset, neostigmine is appropriate only for shallow levels of block and should not be relied upon for rapid reversal. Extubation should be delayed until adequate recovery is confirmed, ideally with quantitative demonstration of TOFR ≥ 0.9 prior to transfer from the operating room or discharge from the PACU [43]. For RNMB after aminosteroidal agents such as rocuronium or vecuronium, sugammadex (2–4 mg kg^−1^) provides rapid and reliable reversal across a wider range of block depths. In infants and neonates, pharmacokinetic variability warrants cautious dosing and post‐reversal observation, as case reports of reappearance of neuromuscular block demonstrate that under‐dosing without proper monitoring can precipitate late weakness. Repeat dosing should be considered if quantitative monitoring confirms persistent block [12]. In settings where objective monitoring is unavailable, any clinical suspicion of RNMB, manifested by inadequate tidal volumes, poor head lift, airway obstruction, or delayed emergence, should prompt empirical reversal with the appropriate agent and continued ventilatory support until full recovery is clinically evident. When immediate reversal is impractical or incomplete, supportive care remains essential: ensure airway patency, maintain oxygenation with positive‐pressure ventilation or continuous positive airway pressure, and continue respiratory support until complete neuromuscular recovery is achieved.

### 8.4 Prevention and Best Practice

The persistence of RNMB in pediatric anesthesia reflects both educational and systemic deficiencies. Despite clear evidence and guidelines, many clinicians continue to depend on unreliable clinical indicators instead of quantitative monitoring, and younger practitioners may over‐rely on agents such as sugammadex, overlooking essential principles of reversal management [13]. Quantitative monitoring devices remain underused due to limited availability, technical challenges in small children, and workflow incompatibilities, particularly with AMG requiring free thumb movement. Inadequate equipment, inconsistent site selection, and financial barriers further hinder standardized monitoring, perpetuating unsafe variability in practice despite clear evidence and professional recommendations [4].

## 9 Summary

Safe neuromuscular block management in pediatric anesthesia requires the integration of pharmacological precision, physiological understanding, and system‐wide vigilance (Table 3). The analysis of the four error traps highlights that adverse outcomes related to NMBA seldom arise from the agents themselves but rather from human, cognitive, and organizational deficiencies throughout their administration, monitoring, and reversal.

**TABLE 3:** Key recommendations for safe neuromuscular block management in children.

Domain	Recommendation
Indication	Administer NMBA when clinically appropriate to optimize intubation conditions and controlled ventilation, particularly in infants and high‐risk airways.
Monitoring	Always employ quantitative monitoring (preferably EMG or AMG) to guide timing and confirm recovery.
Reversal strategy	Match the reversal agent and dose to the NMBA type and block depth.
Neostigmine	Use only after spontaneous recovery (TOFR ≥ 0.4).
Sugammadex	Administer 2–4 mg·kg^−1^ according to block depth.
Decision‐making	Avoid time‐based reversal. Decisions should be guided by objective evidence of recovery.
Extubation	Confirm TOFR ≥ 0.9 before extubation.
Postoperative care	Observe high‐risk patients (infants, renal impairment, heavy NMBA dosing) for early signs of respiratory compromise.
Systems approach	Promote education and standardization through institutional policy, simulation, and audit feedback.

*Note:* TOFR ≥ 0.9 indicates adequate recovery from neuromuscular block.

Abbreviations: AMG, acceleromyography; EMG, electromyography; NMBA, neuromuscular blocking agent; TOFR, train‐of‐four ratio.

From induction to recovery, patient safety depends on a continuous, closed‐loop process encompassing assessment, administration, monitoring, and verified reversal of NMBA. Three domains are particularly critical:

1. Quantitative Monitoring Across the Perioperative Continuum
  Subjective assessment and qualitative peripheral nerve stimulation are insufficient to ensure recovery. Quantitative monitoring must be implemented routinely for all children receiving NMBA. Objective measurement of the TOFR provides the only reliable means of confirming adequate recovery (TOFR ≥ 0.9) and preventing RNMB. This standard should be considered as indispensable as pulse oximetry or capnography in modern practice.
2. Pharmacological Accuracy and Individualized Reversal
  Both neostigmine and sugammadex are effective only when used within their pharmacological limitations and guided by objective assessment of block depth. Pediatric patients, particularly neonates and infants, display significant interindividual variability in pharmacokinetics and pharmacodynamics, necessitating dose adjustment and post‐reversal vigilance.
3. Institutional Readiness and Educational Reinforcement
  Persistent reliance on clinical judgment and time‐based reversal reflects systemic rather than individual shortcomings. Institutional protocols should mandate quantitative monitoring and ensure the availability of appropriate equipment and reversal agents in all operating rooms and recovery areas. Departments seeking to implement quantitative neuromuscular monitoring should frame equipment acquisition as a patient‐safety initiative aligned with established quality metrics, such as reduction in postoperative respiratory complications and unplanned PACU admissions. Presenting local audit data, benchmarking against international guidelines, and embedding neuromuscular monitoring within structured quality‐improvement programs may strengthen institutional support and resource allocation. Engagement with surgical colleagues is equally essential to facilitate limb accessibility when feasible, as safe monitoring depends on interdisciplinary cooperation. Training programs must explicitly address pediatric‐specific aspects of NMBA use, monitoring, and reversal, incorporating simulation, standardized protocols, and audit feedback to reinforce quantitative interpretation as a core competency and to support sustained cultural change.

The principle governing neuromuscular safety in pediatric anesthesia is therefore unambiguous:

No NMBA should ever be administered without a plan and an objective mechanism to ensure their complete and verified reversal. Every child receiving a NMBA must be quantitatively confirmed to have recovered before leaving the operating room or PACU.

Embedding this principle into institutional policy and clinical culture represents a measurable and achievable step towards reducing avoidable perioperative morbidity and advancing the reliability and safety of pediatric anesthetic care.

## Funding

The authors have nothing to report.

## Ethics Statement

The authors have nothing to report.

## Consent

The authors have nothing to report.

## Conflicts of Interest

V.C.Q. is an Editor of *Pediatric Anesthesia*. V.C.Q. receives research funding from the São Paulo Research Foundation (FAPESP; grant no. 2024/07012–9) and from Smile Train Inc. (grant IDs 0206904 and D0198488). D.F. has received honoraria from Senzime for speaking activities. The other authors declare no conflicts of interest.

## Data Availability

The authors have nothing to report.
